# The Mediterranean diet reduces the genetic risk of chromosome 9p21 for myocardial infarction in an Asian population community cohort

**DOI:** 10.1038/s41598-019-54938-w

**Published:** 2019-12-05

**Authors:** Hsin-Bang Leu, Chia-Min Chung, Jaw-Wen Chen, Wen-Harn Pan

**Affiliations:** 1 Institute of Clinical Medicine and Cardiovascular Research Center, National Yang-Ming University, Taipei, Taiwan; 2 Healthcare and Service Center, Taipei Veterans General Hospital, Taipei, Taiwan; 3 Division of Cardiology, Department of Medicine, Taipei Veterans General Hospital, Taipei, Taiwan; 4 Environment-Omics-Diseases Research Centre, China Medical University Hospital, Taichung, Taiwan; 5 Institute of Pharmacology, National Yang-Ming University, Taipei, Taiwan; 6 Department of Medical Research, Taipei Veterans General Hospital, Taipei, Taiwan; 7 Institute of Biomedical Sciences, Academia Sinica, Taipei, Taiwan; 8 Graduate Institute of Biomedical Sciences, China Medical University, Taichung, Taiwan

**Keywords:** Cardiology, Cardiovascular diseases

## Abstract

The interaction of genetic susceptibility and dietary habits in cardiovascular disease (CVD) remains undetermined. The purpose of this study was to investigate whether a Mediterranean dietary style modified the genetic risk of developing CVD in a Chinese cohort. A total of 2098 subjects with dietary information from a Chinese community cohort (CVDFACTS) were enrolled. Candidate genes, including SNP markers rs1333049 (*CDKN2B*, 9p21.3), rs17465637 (*MIA3*, 1q41) and rs501120 (*CXCL12*, 10q11.21), were genotyped to analyze the association with future CVD. The impact of dietary pattern was also analyzed according to adherence to the diet using the Mediterranean Diet Score (MDS). After an average follow-up of 7.8 years, only the C risk allele of rs1333049 at chromosome 9p21.3 was associated with a higher risk of MI with either an additive [HR = 1.78, 95% CI:1.23–2.5] or a recessive model [HR = 2.40, 95% CI: 1.42–4.04], and the CC genotype had a higher risk of developing MI (p = 0.009, log-rank test). There was no significant difference in the association of the lipid profile with future CV outcomes among the MDS tertiles. However, the high MI risk of the CC genotype in individuals consuming a less healthy diet (MDS1) (HR: 6.39, 95% CI: 1.74–23.43) significantly decreased to 2.38 (95% CI: 0.57–10.04) in individuals consuming a healthier diet (MDS3), indicating that a healthier dietary pattern (higher MDS) modified the risk of developing MI in carriers of variants in *CDKN2B*. In conclusion, genetic variants of *CDKN2B* at 9p21 were significantly associated with future MI risk in a Chinese cohort, and the genetic risk of MI could be modified by a healthier diet.

## Introduction

Cardiovascular disease (CVD) is associated with an increased risk of mortality and morbidity^1^. Although great effort has been made to prevent disease progression, the occurrence of adverse events, including myocardial infarction, ischemic stroke and cardiovascular death, remains a concern. Atherosclerosis is the central component in the pathogenesis of CVD and is considered a sustained inflammatory process comprising interactions among vascular inflammation, lipid accumulation, oxidative stress, and genetic susceptibility^2^. In addition to medical treatment, lifestyle modifications, especially healthy diets, are encouraged for CVD prevention. Among various CVD risk factors, genetic susceptibility is believed to play some role in determining future CV outcomes. However, there is limited information about the association between genetic risk and dietary habits to CVD.

The importance of dietary patterns to reduce CVD risk has been emphasized in several primary prevention studies for CVD^3,4^. Dietary recommendations of reducing fat intake, replacing saturated fats (cream, butter, cheese) and trans fats with unsaturated fats and increasing the intake of vegetables, fruits, and fish are suggested to prevent adverse CV events^5^. Adherence to different healthy diet patterns, such as the Mediterranean diet (MD)^5^ or the Dietary Approaches to Stop Hypertension (DASH) diet^3^, are reported to be associated with a lower risk of developing cardio-metabolic disorders, suggesting the benefit of a healthy dietary pattern for cardiovascular system protection. To our interest, dietary components differ across different geographic areas, cultures, and environmental exposures and may exert variable impacts in populations with different genetic backgrounds. A previous genome-wide association study has reported several significant associations with CVD, including *CDKN2B* (chromosome 9p21.3), MIA3 (1q41) and *CXCL12* (10q11.21)^6,7^, but the association in the Asian population was not verified using a long-term follow-up cohort. In addition, the impact of a healthy diet on the future risk of cardiovascular disease in the Asian population was unknown. Therefore, it is important to investigate the interactions between the genetic risk for CVD and diet and whether healthy dietary patterns could modify the genetic risk for CVD in the Chinese population. Our current study aimed to investigate the genetic risk of developing CVD and the impact of healthy Mediterranean dietary patterns in a community Chinese cohort.

## Methods

### Study population

The study cohort was selected from the CardioVascular Disease risk FACtors Two-township Study (CVDFACTS), and the study protocol has been reported previously^8,9^. Briefly, the CVDFACTS was a community-based follow-up study investigating CVD occurrence and risk factors in Taiwan since 1989. Five villages with more than 1000 people and a population density greater than 200 persons per square kilometer were randomly selected from Chu-Dong (northwest Taiwan) and Pu-Tzu (southwest Taiwan). Data regarding lifestyle, CVD risk factors, a history of CVD, and urine and blood chemistry data were collected. Repeated examinations were carried out in 1989–1990, 1990–1993, 1994–1997, 1997–1999, and 2000–2002. A total of 5146 residents aged 30 years and older participated in the cycle-3 examination (1994–1996), in which biochemical data were collected; 2098 individuals with complete dietary information were enrolled. The study complied with the Declaration of Helsinki and was approved by the appropriate Ethics Committees of Academia Sinica, and all patients all provided written informed consent at baseline and follow-up visits.

### Blood sampling and genetic markers associated with CVD

Blood samples were collected after an overnight fast. The plasma and buffy coat were separated by centrifugation, and all samples were stored at −70 °C. Biochemical profiles, including glucose, cholesterol, triglycerides, low-density lipoprotein cholesterol (LDL-C), and high-density lipoprotein cholesterol (HDL-C), were measured using a Monarch 2000 automatic analyzer. Genetic susceptibility may interact with environmental factors such as diet, sedentary habits, and smoking exposure to contribute to the complexity of CVD^1^. We studied 3 loci (1q41, 9p21.3, 10q11.21) that have been linked to CAD risk by previous GWAS^6,7^. We genotyped the 3 lead SNPs within the following loci: chromosome 9p21.3 (rs1333049, *CDKN2B*), 1q41 (rs17465637, *MIA3*) and 10q11.21 (rs501120, *CXCL12*)^6,7^. Genotyping was carried out using matrix-assisted laser desorption ionization time-of-flight (MALDI-TOF) mass spectrometry (Sequenom, MassARRAY, San Diego, CA, USA) using standard protocols and was performed at the Academia Sinica National Genotyping Center, Taiwan. Automated genotype calling was performed, and the data were analyzed using Sequenom Typer software.

### Mediterranean dietary pattern

Dietary intake information was assessed using a food-frequency questionnaire that included foods and beverages commonly consumed in Taiwan. The Mediterranean Diet Score (MDS) was calculated based on components as reported previously^5,10^. For each of the dietary items, participants were asked to report the frequency of consumption and portion size. In brief, a total of 14 inclusive food groups, including vegetables, fruits, nuts, legumes, cereals, meats, dairy products, fish, eggs, monounsaturated lipids (e.g., olive oil), polyunsaturated lipids, saturated lipids, sugar, and sweetened and non-alcoholic beverages, were included. Each question was scored 0 or 1, and a higher MDS indicated greater adherence to the Mediterranean dietary pattern^5,10^.

### Clinical follow-up for adverse CV events

Cardiovascular events, including ischemic stroke, fatal or nonfatal MI, and CV death, were identified from a review of self-reported disease histories, death certificates, and insurance claim records in the National Health Insurance (NHI) database dated until the end of 2002. The NHI included 99.5% of Taiwan’s population. The CV events were identified from NHI database records using codes 430 to 438 for ischemic stroke and 410 to 414 for CAD from the International Classification of Diseases, 9th Revision, Clinical Modification (ICD-9-CM). Cardiovascular death was identified from death certificates. We used the National Death Registry database, which obtains information from certified death certificates and codes death according to the ICD-9. The sensitivity and specificity for event identification were 100% and 95%, respectively^9,11,12^.

### Statistical analysis

All baseline data were expressed as either the mean ± standard deviation (SD) or number (percentage). SPSS statistical software version 10.0 (Chicago, IL) was used for statistical analysis. The significance of between-group differences in means was assessed using Student’s t-test, and the significance of differences between 2 proportions was tested with the chi-squared test. SNPs were excluded when the SNP call rate was<95% or not in Hardy–Weinberg equilibrium. Associations between SNPs and future adverse outcomes were evaluated in three models (additive, dominant, and recessive) after adjusting for covariates. Data on outcomes were censored either at the time of an ischemic stroke event or death or at the end of follow-up. Test for trend was also carried out by assigning the ordinal value 0, 1, and 2 according to the numbers of risk alleles and then examining the association of the resulting variable with the outcome. Kaplan–Meier curves with log-rank tests for event-free survival were constructed for genetic variants and dietary patterns as described previously^13,14^. To determine the hazard ratio (HR) of the genetic variant, a Cox model was performed. The results were presented as the HR with its corresponding 95% confidence interval (CI). A p value < 0.05 was considered statistically significant.

### Ethics approval and consent to participate

The study complied with the Declaration of Helsinki, which was approved by the appropriate Health Authorities, independent Ethics Committees, and Independent Review Boards of Academia Sinica. All patients should give their written inform consent before enrollment.

## Results

A total of 2,098 subjects, 917 males and 1181 females with a mean age of 49.9 ± 12.2 years, with complete dietary information were included in this study. Their baseline characteristics are shown in Table 1. The proportion with hypertension and that of smokers was 12.4% and 19.3%, respectively, and the proportion with diabetes was 3.1%. The BMI was 24.2 ± 3.3 kg/m^2^. Because study subjects were enrolled from the community and not from hospitals, the lipid profiles were relatively normal (LDL-C: 130.5 ± 44.5 mg/dL; HDL: 42.7 ± 15.5 mg/dL; triglycerides: 107.7 ± 76.5 mg/dL, respectively). The genotype call rate was above 99.4%, and the mean MDS was 5.1 ± 1.2 with normal distribution (Supplement Fig. 2). Frequencies of the candidate SNP markers, including rs1333049 (*CDKN2B*, 9p21.3), rs17465637 (*MIA3*, 1q41), and rs501120 (*CXCL12*, 10q11.21), are shown in Table 1.

**Table 1:** Baseline characteristics of subjects with future cardiovascular events in community-based cohort study.

	Total subjects	Subjects developing ischemic stroke	Subjects developing MI	Subjects developing CV death	Subjects developing total events
	(n = 2098)	(n = 54)	(n = 64)	(n = 26)	(n = 134)
Age, year	49.9 ± 12.2	57.9 ± 8.7	58.9 ± 9.2	64.9 ± 7.8	59.4 ± 9.3
Male, n(%)	917 (43.7)	29 (53.7)	35 (54.7)	17 (65.4)	75 (56.0)
Hypertension, n(%)	260 (12.4)	15 (27.8)	26 (40.6)	7 (26.9)	44 (32.8)
Diabetes, n(%)	65 (3.1)	6 (11.1)	8 (12.5)	3 (11.5)	14 (10.4)
Smoking, n(%)	405 (19.3)	15 (27.8)	21 (32.8)	9 (34.6)	40 (29.9)
Systolic BP, mmHg	119.0 ± 18.2	131.4 ± 22.4	132.3 ± 21.4	130.7 ± 24.8	131.2 ± 22.5
Diastolic BP, mmHg	74.1 ± 11.1	80.7 ± 9.7	78.9 ± 12.2	75.6 ± 8.4	78.9 ± 11.0
BMI, Kg/m^2^	24.2 ± 3.3	25.1 ± 4.3	25.1 ± 3.5	23.1 ± 3.7	24.8 ± 4.0
Waist, cm	80.4 ± 9.4	84.1 ± 11.5	86.1 ± 10.0	81.8 ± 11.0	84.4 ± 10.7
Triglyceride, mg/dL	107.7 ± 76.5	124.9 ± 80.7	139.6 ± 83.1	108.3 ± 48.0	128.6 ± 79.2
Total cholesterol, mg/dL	196.5 ± 42.9	201.2 ± 43.8	212.7 ± 42.1	194.8 ± 35.7	204.9 ± 43.0
HDL-C, mg/dL	42.7 ± 15.5	40.6 ± 9.9	39.1 ± 11.2	43.4 ± 12.2	40.3 ± 11.0
Glucose, mg/dL	99.5 ± 24.7	107.0 ± 29.4	115.0 ± 52.5	110.3 ± 47.2	110.1 ± 41.3
LDL-C, mg/dL	130.5 ± 44.5	123.3 ± 40.9	136.9 ± 39.6	122.3 ± 27.9	128.6 ± 39.3
High-sensitive CRP, mg/L	0.98 (0.09–253.74)	1.57 (0.20–22.08)	1.26 (0.22–18.85)	1.28 (0.44–6.27)	1.55 (0.20–22.08)
MDS score	5.1 ± 1.2	4.9 ± 1.3	5.1 ± 1.1	5.3 ± 1.2	5.0 ± 1.2
**rs1333049 genotype**					
GG	584 (27.8)	15 (27.8)	12 (18.8)	6 (23.1)	31 (23.1)
GC	1070 (51.0)	30 (55.6)	30 (46.9)	15 (57.7)	71 (53.0)
CC	444 (21.2)	9 (16.7)	22 (34.4)	5 (19.2)	32 (23.9)
**rs17465637 genotype**					
CC	825 (39.7)	23 (42.6)	21 (33.3)	11 (42.3)	52 (39.1)
CA	971 (46.7)	25 (46.3)	34 (54.0)	12 (46.2)	65 (48.9)
AA	283 (13.6)	6 (11.1)	8 (12.7)	3 (11.5)	16 (12.0)
**rs501120 genotype**					
TT	888 (43.2)	21 (40.4)	29 (46.8)	16 (61.5)	60 (46.2)
TC	907 (44.1)	21 (40.4)	28 (45.2)	8 (30.8)	53 (40.8)
CC	262 (12.7)	10 (19.2)	5 (8.1)	2 (7.7)	17 (13.1)

Values Data are n (%) or mean ± SD, BMI indicates body mass index; LDL-C: low density lipoprotein cholesterol; HDL-C, HDL: high density lipoprotein-cholesterol; CRP, C-reactive protein; MI, myocardial infarction; MDS, Mediterranean diet score.

**Figure 1:**
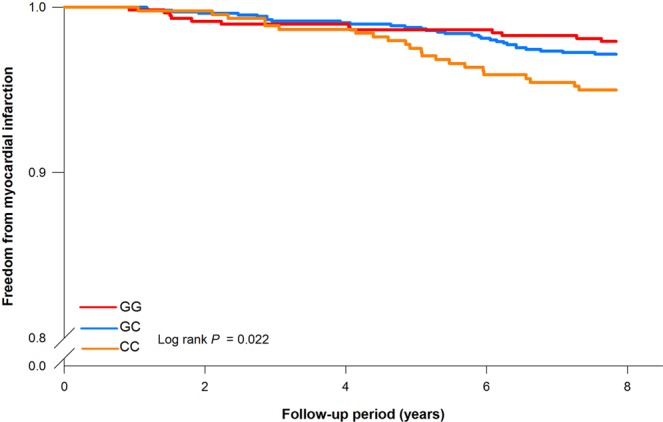
Kaplan-Meier estimates of survival-free of future myocardial infarction according to rs1333049. The CC genotype has significant lower future event-free survival rates for MI (log-rank test, p = 0.022).

After an average of 7.8 years of follow-up, a total of 134 major CV events were identified, including 54 ischemic strokes, 64 acute MIs, and 26 cardiovascular-related deaths (Table 1). Subjects with future CV events were older, more likely to be male, and had a higher prevalence of hypertension, diabetes, and cigarette smoking. There were no significant differences in lipid profiles at baseline between subjects with and without a CV event, but subjects with a CV event had a higher BMI and a higher level of high-sensitivity C-reactive protein (hsCRP), suggesting that an increased inflammatory state was present in those who later developed a CV event. Of the selected genetic SNP markers, only the C risk allele of rs1333049 at chromosome 9p21.3 was associated with an increased risk of future MI occurrence using either an additive (HR = 1.78, 95% CI: 1.23–2.58, p = 0.002) or a recessive (HR = 2.40, 95% CI: 1.42–4.04, p = 0.001) model after adjusting for age, sex, hypertension, diabetes, tobacco smoking, and lipid profile (Table 2). Kaplan–Meier survival analysis also showed that the CC genotype of the rs1333049 variant had a higher future MI risk (p = 0.009 by log-rank test) (Fig. 1). The Supplement Table 1 showed that CC genotype of rs1333049 variant had higher incidence of MI development during follow-up period (Supplement Table 1**)**. There were no significant genetic associations observed among rs17465637 (*MIA3*) or rs501120 (*CXCL12*) and future adverse CV events, including MI, ischemic stroke, CV death and major adverse cardiovascular events (MACEs). The subgroup analysis (Supplement Fig. 1) further showed that the CC genotype of the rs1333049 polymorphism for MI risk was especially observed in females and those <65 years old, with a higher BMI, unfavorable lipid profile, and higher baseline CRP values.

**Table 2:** Association of genetic risk and future CV events.

SNP marker, risk allele	Dominant		Recessive		Additive	
	HR	P-value	HR	P-value	HR	P-value
**rs1333049 genotype, C allele**						
Stroke	1.02 (0.56–1.86)	0.942	0.82 (0.40–1.69)	0.597	0.95 (0.64–1.41)	0.796
CV mortality	1.30 (0.52–3.26)	0.570	1.13 (0.42–3.02)	0.810	1.17 (0.65–2.08)	0.602
Myocardial infarction	1.75 (0.93–3.28)	0.082	**2.40 (1.42–4.04)**	0.001	**1.78 (1.23–2.58)**	**0.002**
Major cardiovascular event	1.34 (0.90–2.01)	0.149	1.39 (0.93–2.07)	0.107	1.27 (0.99–1.63)	0.059
**rs17465637 genotype, A allele**						
Stroke	1.36 (0.58–3.18)	0.481	1.24 (0.72–2.13)	0.444	1.20 (0.81–1.80)	0.365
CV mortality	1.80 (0.52–6.16)	0.350	1.43 (0.65–3.18)	0.377	1.39 (0.78–2.49)	0.264
Myocardial infarction	1.21 (0.57–2.55)	0.616	0.88 (0.52–1.49)	0.627	0.99 (0.68–1.42)	0.938
Major cardiovascular event	1.30 (0.77–2.20)	0.326	1.10 (0.77–1.56)	0.605	1.12 (0.87–1.44)	0.380
**rs501120 genotype, C allele**						
Stroke	1.12 (0.64–1.95)	0.698	1.76 (0.88–3.51)	0.111	0.81 (0.54–1.20)	0.286
CV mortality	0.43 (0.19–1.01)	0.061	0.68 (0.16–2.91)	0.603	1.87 (0.96–3.64)	0.067
Myocardial infarction	0.84 (0.51–1.38)	0.486	0.64 (0.26–1.60)	0.339	1.21 (0.82–1.78)	0.327
Major cardiovascular event	0.86 (0.60–1.21)	0.381	1.09 (0.66–1.82)	0.733	1.07 (0.82–1.38)	0.634

Values Data are n (%) or mean ± SD, BMI indicates body mass index; LDL-C: low density lipoprotein cholesterol; HDL-C, HDL: high density lipoprotein-cholesterol; CRP, C-reactive protein.

### Impact of the Mediterranean dietary pattern on future events

**Figure 2:**
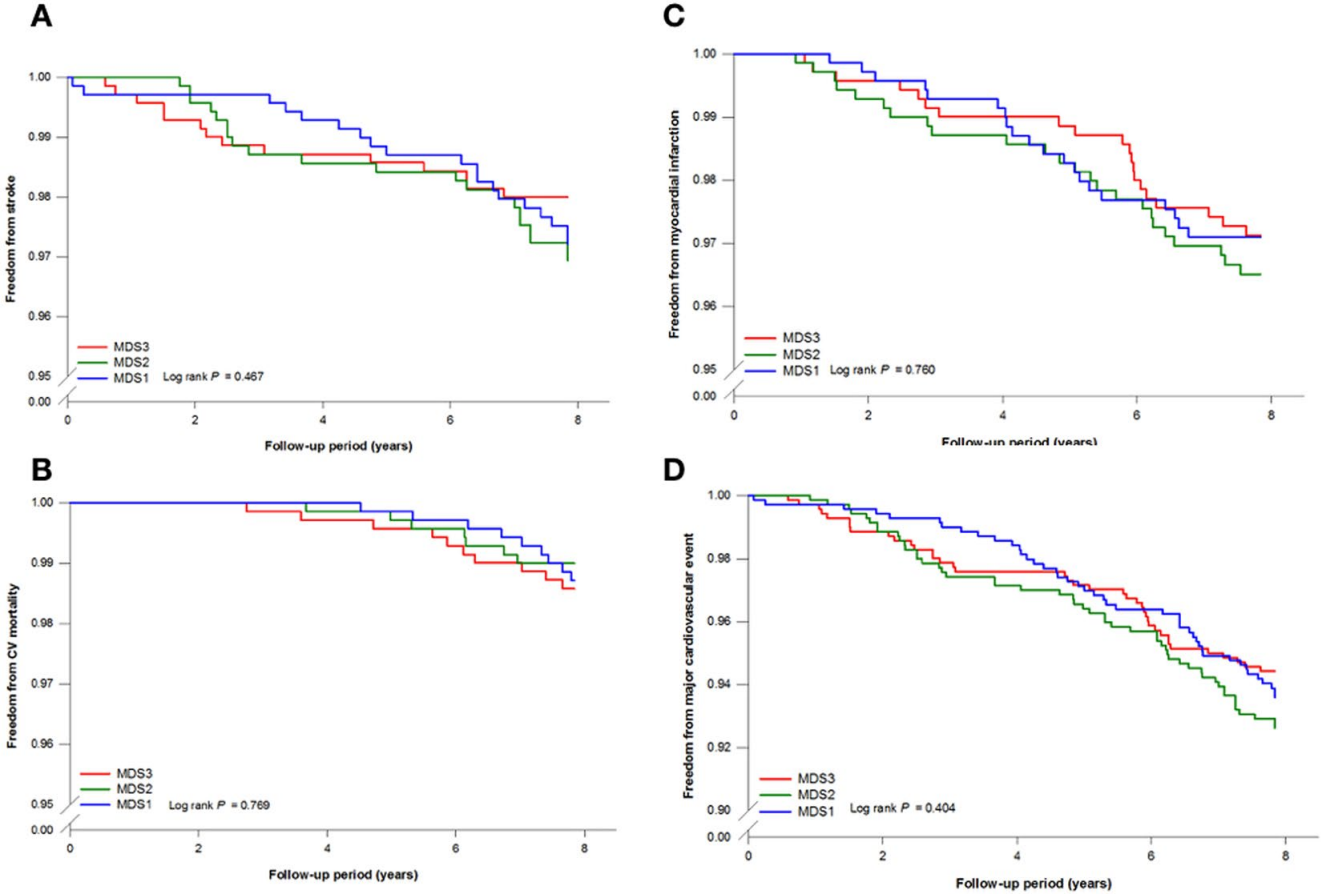
Kaplan-Meier estimates of survival-free cardiovascular events including stroke (**A**), cardiovascular death (**B**), myocardial infarction (**C**) and total cardiovascular events (**D**) according to MDS tertiles in a cohort study. There is no significant association found between MDS and future cardiovascular events.

The impact of diet was analyzed based on the MDS^5,10^ and was categorized as tertiles for the analysis. The distribution of MDS in study population was presented in Supplement Fig. 2. The subject baseline characteristics by MDS tertiles (MDS1, MDS2, MDS3) are shown in Table 3. There were no significant differences in the lipid profiles, body weight, and CVD risk factors among the tertiles of the MDS. Subjects with healthier dietary patterns (MDS2, MDS3) tended to be older and have a higher baseline blood pressure than those with a less healthy dietary pattern (MDS1) (p < 0.05). As also demonstrated in Table 3, the genotype frequency and incidence of CV events were similar among these MDS tertiles. The Kaplan–Meier survival analysis did not show any differences in CV event-survival curves derived for the three MDS tertiles in future events, including MI, ischemic stroke, CV death and MACEs (Fig. 2).

**Table 3:** Baseline characteristics and future CV outcome according to dietary habit by Mediterranean diet score.

	MDS1	MDS2	MDS3	P-value
	(n = 697)	(n = 698)	(n = 703)	
MDS	3.76 ± 0.46	5.00 ± 0.0	6.52 ± 0.77	<0.001
Age, year	48.4 ± 12.7	50.7 ± 12.3	50.6 ± 11.4	<0.001
Male, n(%)	294 (42.2)	324 (46.4)	299 (42.5)	0.208
Hypertension, n(%)	63 (9.0)	92 (13.2)	105 (14.9)	0.003
Diabetes, n(%)	20 (2.9)	17 (2.4)	28 (4.0)	0.226
Smoking, n(%)	140 (20.1)	141 (20.2)	124 (17.6)	0.390
Systolic BP, mmHg	117.5 ± 17.3	119.9 ± 19.0	119.7 ± 18.1	0.024
Diastolic BP, mmHg	72.9 ± 11.0	74.4 ± 11.6	75.1 ± 10.6	0.001
BMI, Kg/m^2^	24.1 ± 3.5	24.2 ± 3.4	24.4 ± 3.1	0.189
Waist, cm	80.0 ± 10.0	80.5 ± 9.2	80.6 ± 9.0	0.439
Triglyceride, mg/dL	105.6 ± 75.4	114.0 ± 87.0	103.7 ± 65.7	0.041
Total cholesterol, mg/dL	196.1 ± 41.5	197.3 ± 43.2	196.1 ± 43.9	0.860
HDL-C, mg/dL	43.2 ± 13.2	43.1 ± 19.7	41.9 ± 12.3	0.375
Glucose, mg/dL	100.6 ± 29.3	98.8 ± 21.3	98.9 ± 22.9	0.331
LDL-C, mg/dL	129.3 ± 34.5	131.0 ± 56.4	131.2 ± 38.7	0.785
High-sensitive CRP, mg/L	0.96 (0.11–253.74)	1.00 (0.1–102.90)	0.96 (0.09–38.68)	0.827
rs1333049 genotype				0.463
GG	188 (27.0)	197 (28.2)	199 (28.3)	
GC	350 (50.2)	366 (52.4)	354 (50.4)	
CC	159 (22.8)	135 (19.3)	150 (21.3)	
rs17465637 genotype				0.043
CC	294 (42.4)	276 (39.9)	255 (36.7)	
CA	321 (46.3)	324 (46.9)	326 (46.9)	
AA	78 (11.3)	91 (13.2)	114 (16.4)	
rs501120 genotype				0.851
TT	284 (41.7)	305 (44.3)	299 (43.5)	
TC	309 (45.4)	301 (43.7)	297 (43.2)	
CC	88 (12.9)	83 (12.0)	91 (13.2)	
Stroke	19 (2.7)	21 (3.0)	14 (2.0)	0.463
CV mortality	9 (1.3)	7 (1.0)	10 (1.4)	0.768
Myocardial infarction	20 (2.9)	24 (3.4)	20 (2.8)	0.766
Major cardiovascular event	44 (6.3)	51 (7.3)	39 (5.5)	0.402

Values Data are n (%) or mean ± SD, BMI indicates body mass index; LDL-C: low density lipoprotein cholesterol; HDL-C, HDL: high density lipoprotein-cholesterol; CRP, C-reactive protein; MDS, MDS, Mediterranean diet score.

**Figure 3:**
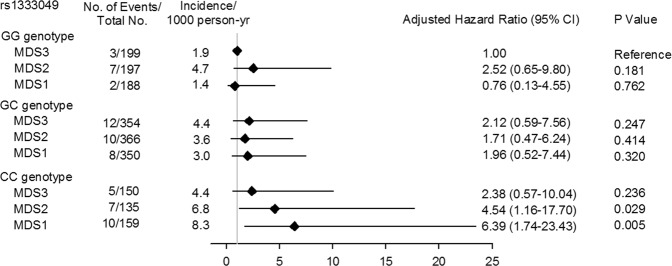
The association between dietary MDS and genetic risk (rs1333049) of developing future MI. The MI risk in CC genotype in the 1^st^ MDS tertile decreased in the 3^rd^ MDS tertile (healthier dietary pattern)(HR: 2.38, 95%CI:0.57–10.04) compared with the GG genotype in MDS3 (referent).

To investigate the interaction between the Mediterranean diet and the genetic risk of CVD, the hazard ratio (HR) of rs1333049 genotypes for MI risk according to MDS tertiles was examined. Although the MDS did not independently affect CV outcome in our study, the high MI risk of the CC genotype in the less healthy diet pattern (MDS1) (HR: 6.39, 95% CI: 1.74–23.43) significantly decreased to 2.38 (95% CI: 0.57–10.04) in the healthier dietary pattern (MDS3) (Fig. 3), while this phenomenon was not seen for those with the GG genotype, indicating that the genetic risk for MI could be modified by a healthier diet and that the effect of risk genes may not be evident if a healthy dietary pattern is maintained.

## Discussion

The current study showed that the rs1333049 polymorphism at 9p21 was a genetic determinant for future MI in a Chinese population and that individuals with the CC genotype have a higher risk of developing an MI. Regarding dietary impact on CVD, although there was no significant association between the MDS and future CV risk, the risk of the C allele of rs1333049 at 9p21 for MI can be blunted by a Taiwanese Mediterranean-like diet (higher MDS), indicating that the genetic risk of developing MI may be modified by a healthier diet and supporting the notion that a favorable diet should be recommended for CV event prevention in Asian populations.

Most association studies of the genetic variants and CHD was the association between genetic markers and the prevalence of disease, and the association was inconsistent. The reason why there no association of 1q419 (rs17465637, MIA3) and 10q11.21 (rs501120, CXCL12) with future events was observed may come from the difference in various ethnicities or various CHD definitions (myocardial infarction, coronary angioplasty or bypass graft). This polymorphism (rs501120, CXCL12) has been studied in several populations. In Caucasians, this locus was found to be associated with coronary artery disease^8,15^ in large-scale association studies. However, Haver *et al*. failed to replicate the association of this locus with CVD events^16^. Inconsistency is also noted for the region of 1q419 (rs17465637, MIA3); this locus was associated with CAD identified by the GWAS study but was not in subsequent studies. Wang *et al*. reported the association of this SNP with CAD in American Caucasians^17^, whereas the association remained inconsistent in follow-up studies on Chinese people^18,19^. However, a subsequent meta-analysis on five Asian populations confirmed the association with CAD in this ethnic group^20^. The association of the genetic risk of variants of *CDKN2B* at 9p21 and CAD has been reported previously^6^. Although the C allele in rs1333049 has been identified as a risk allele having the strongest association with CAD in both the WTCCC and the German GWAS^6^, the associations between this loci and CAD were not consistent^14^. Buysschaert *et al*. reported that the at-risk C allele at rs1333049 was significantly and independently associated with recurrent MI and cardiac death^13^. Additionally, the C allele at rs1333049 was reported to be associated with a lower risk of re-infarction in patients after coronary intervention^21^. In this study, we demonstrated that the C allele at rs1333049 was associated with a risk of MI in an Asian general population using a prospective observation design. Lee *et al*. reported that rs4977574 at 9p21 is associated with CAD in Han Chinese but did not predict CV mortality^22^. A prospective study of a multiethnic cohort, which is similar to ours investigating the same SNP markers, 9p21.3 (rs1333049; *CDKN2B*), 1q419 (rs17465637, *MIA3*) and 10q11.21 (rs501120, *CXCL12*), reported 9 loci, including the 9p21 region, and CXCL12 (rs501120), which were statistically associated with incident CHD events in the white population but not in the Asian/Pacific population, and only the 9p21 region showed significant between-study heterogeneity^23^. Furthermore, the association between a SNP in the 9p21 region and incident CHD was replicated in Asian/Pacific ethnicity later, but no significant association between 1q419 (rs17465637, *MIA3*) or 10q11.21 (rs501120, *CXCL12*) and CHD was observed in the Asian population in this replication study^23^. The association of the 9p21 region with CHD has also been replicated in Asian populations, including Pakistani^23,24^, Japanese and Korean^23,25,26^ and Han Chinese^23,27^ populations, suggesting that the 9p21 region is the most robust common genetic risk factor in determining the risk of future CHD. Our current finding extended previous observations using a prospective cohort study that yielded causal evidence because exposure precedes the development of the disease^28^. Our study confirmed the association of 9p21 and CAD and the importance of the C allele of rs1333049 in predicting MI risk in the general population.

The Mediterranean diet, which is high in vegetables, fruit, fish, whole grains, red wine, and olive oil, has demonstrated protective effects toward multiple noncommunicable diseases. Recent studies of consuming a Mediterranean-style diet showed a reduction in CV events even to a greater degree than that of low-fat diets and a benefit equal to or greater than that of statin therapies^8,9,11,12^. The MDS indicates the degree of adherence to the Mediterranean diet^29^, and it has been used to evaluate the impact of the diet in determining CV risk and survival^30^. The Taiwanese diet in the 1990s included MD components such as vegetables, fruits, and fish but not olive oil, whole grains, or red wine. Instead, Taiwanese individuals used soybean oil or lard for cooking, consumed mostly white rice as a staple, and drank rice wine for social events. Our current study did not show a significant benefit of a Taiwanese MD-like diet (higher MDS) in CVD prevention. Nonetheless, subjects with a higher MDS tended to have favorable lipid profiles, although the difference between tertiles was not significant.

In our study, the risk of MI was greatest in individuals with the CC genotype of rs1333049 who consumed an unhealthy diet (lower MDS), but the risk was significantly reduced if individuals with the CC genotype of rs1333049 adhered to a healthier diet (higher MDS), indicating that the MD-like diet modified the genetic association of rs1333049 at 9p21 with the risk of MI, and the effect of the risk gene may not be evident if a healthy dietary pattern is maintained. Similar observations of genetic risk modified by lifestyle or healthy diet have been reported. Corella *et al*. first reported that a higher adherence to a Mediterranean-style diet attenuated the genetic risk of increased blood sugar in individuals with the TT genotype of rs7903146 in the *TCF7L2* gene^10^. Furthermore, the higher stroke risk of the TT genotype shown in the control group (HR = 3.06, 95% CI: 1.43–6.59) was not observed in Mediterranean-style diet intervention groups after a 4.8-year follow-up^10^. Similarly, Zheng *et al*., who investigated the association between sugar-sweetened beverage (SSB) intake and MI risk in a Hispanic population with chromosome 9p21 variants, showed that the per–risk allele odds ratio (OR) of MI in subjects with the rs4977574 polymorphism was 1.44 in subjects with higher SSB consumption (>2 servings/day), 1.21 in subjects with average consumption (1–2 servings/day), and 0.97 in subjects lower consumption (<1 serving/d)^31^. This finding suggested that higher SSB intake could exacerbate the harmful effects of chromosome 9p21 variants on CAD and was consistent with our finding that genetic risk could be modified by diet.

There are some limitations of this study that should be taken into consideration. The mechanisms by which a healthy diet decreases the risks associated with the rs4977574 polymorphism remain undetermined. Several mechanisms have been proposed, including epigenetic regulation of a healthy diet changing methylation levels to a favorable profile in carriers of the risk allele. In addition, DNA methylation, histone modification and microRNA modulation affected by diet have also been mentioned^32^. Finally, many healthy eating behaviors co-occur with other healthy lifestyle behaviors, such as regular exercise, and these behaviors may confound the dietary results. Our current study results were in accordance with a recently published study that showed that a favorable lifestyle was associated with a nearly 50% decrease in the risk of CAD in patients at higher genetic risk^33^.

In conclusion, the association of the rs1333049 polymorphism at 9p21 with the risk of MI was confirmed in a Chinese cohort population, and a healthy Taiwanese MD-like diet attenuated its genetic risk for future MI.

## Supplementary information

supplementary table and figures
