# Supplementary material for: The Mediterranean diet reduces the genetic risk of chromosome 9p21 for myocardial infarction in an Asian population community cohort
Source: Sci Rep. 2019 Dec 5;9:18405. doi: 10.1038/s41598-019-54938-w (PMC6895036; doi:10.1038/s41598-019-54938-w)

The Mediterranean diet reduces the genetic risk of chromosome 9p21 for myocardial infarction in an Asian population community cohort

*Hsin-Bang Leu,[1,2,3] Chia-Min Chung,[4] Jaw-Wen Chen,[1,3,5,6] *Wen-Harn Pan[7]

[1]Institute of Clinical Medicine and Cardiovascular Research Center, National Yang-Ming University, Taipei, Taiwan

[2] Healthcare and Service Center, Taipei Veterans General Hospital, Taipei, Taiwan

[3] Division of Cardiology, Department of Medicine, Taipei Veterans General Hospital, Taipei, Taiwan

[4] China Medical University

[5]Institute of Pharmacology, National Yang-Ming University, Taipei, Taiwan

[6]Department of Medical Research, Taipei Veterans General Hospital, Taipei, Taiwan

[7]Institute of Biomedical Sciences, Academia Sinica, Taipei, Taiwan

＊ Hsin-Bang Leu and Wen-Harn Pan contributed equally to this work and both are corresponding authors of this paper

Hsin-Bang Leu, MD, PhD, FACC

Healthcare and Service Center

Division of Cardiology, Taipei Veterans General Hospital, Taipei, Taiwan

201 Sec. 2, Shih-Pai Road, Taipei, Taiwan.

E-mail: hsinbangleu@gmail.com, pan@ibms.sinica.edu.tw

TEL: +886-28712121 ext.3470

Supplementary table 1 Baseline characteristics and future cardiovascular events during follow-up period according to genotype of rs1333049

	GG (N=584)	GC (n=1070)	CC (n=444)	P-Value
Age, year	50.2 ± 12.1	50.1 ± 12.4	49.0 ± 12.0	0.226
Male, n(%)	274 (46.9)	473 (44.2)	170 (38.3)	0.020
Hypertension, n(%)	72 (12.3)	139 (13.0)	49 (11.0)	0.575
Diabetes, n(%)	13 (2.2)	35 (3.3)	17 (3.8)	0.305
Smoking, n(%)	129 (22.1)	205 (19.2)	71 (16.0)	0.049
Systolic BP, mmHg	118.8 ± 18.9	118.9 ± 18.1	119.6 ± 17.6	0.742
Diastolic BP, mmHg	74.3 ± 11.4	74.1 ± 11.0	74.1 ± 10.9	0.960
BMI, Kg/m^2	24.3 ± 3.2	24.2 ± 3.4	24.3 ± 3.1	0.819
Waist, cm	80.6 ± 9.4	80.3 ± 9.4	80.4 ± 9.4	0.834
Triglyceride, mg/dL	108.2 ± 71.7	108.7 ± 77.7	104.7 ± 79.8	0.679
Total cholesterol, mg/dL	196.7 ± 42.2	196.9 ± 43.5	195.2 ± 42.5	0.798
HDL-C, mg/dL	42.4 ± 20.1	43.0 ± 13.6	42.4 ± 12.6	0.821
Glucose, mg/dL	98.7 ± 22.4	99.4 ± 25.1	100.5 ± 26.8	0.510
LDL-C, mg/dL	129.5 ± 36.8	129.7 ± 38.0	134.1 ± 64.7	0.335
High-sensitive CRP, mg/L	1.10 (0.11-211.76)	0.96 (0.10-32.97)	0.90 (0.09-253.74)	0.119
Stroke	15 (2.6)	30 (2.8)	9 (2.0)	0.686
CV mortality	6 (1.0)	15 (1.4)	5 (1.1)	0.782
Myocardial infarction	12 (2.1)	30 (2.8)	22 (5.0)	**0.022**
Major cardiovascular event	31 (5.3)	71 (6.6)	32 (7.2)	0.418

Values Data are n (%) or mean ± SD, BMI indicates body mass index; LDL-C: low density lipoprotein cholesterol; HDL-C, HDL: high density lipoprotein-cholesterol; CRP, C-reactive protein; MI, myocardial infarction

Figure legends

Supplement figure 1. Subgroup analysis of the association of genotype of rs1333048 and MI risk

Supplement figure 2. Distribution of MDS in study subjects

Supplementary figure 1 Subgroup analysis of the association of genotype of rs1333048 and MI risk

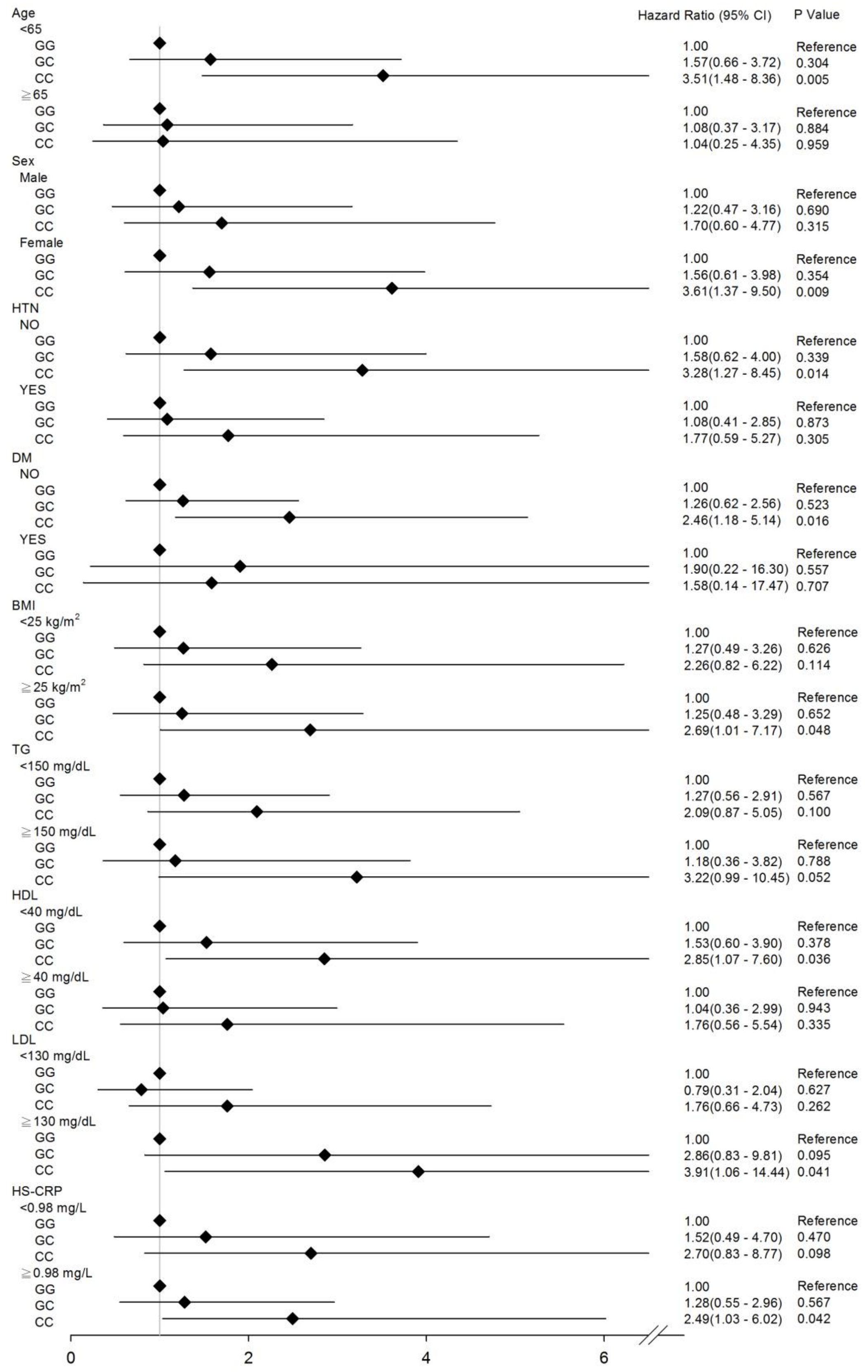

Supplementary figure 2
Distribution of MDS in study subjects (number of subjects)

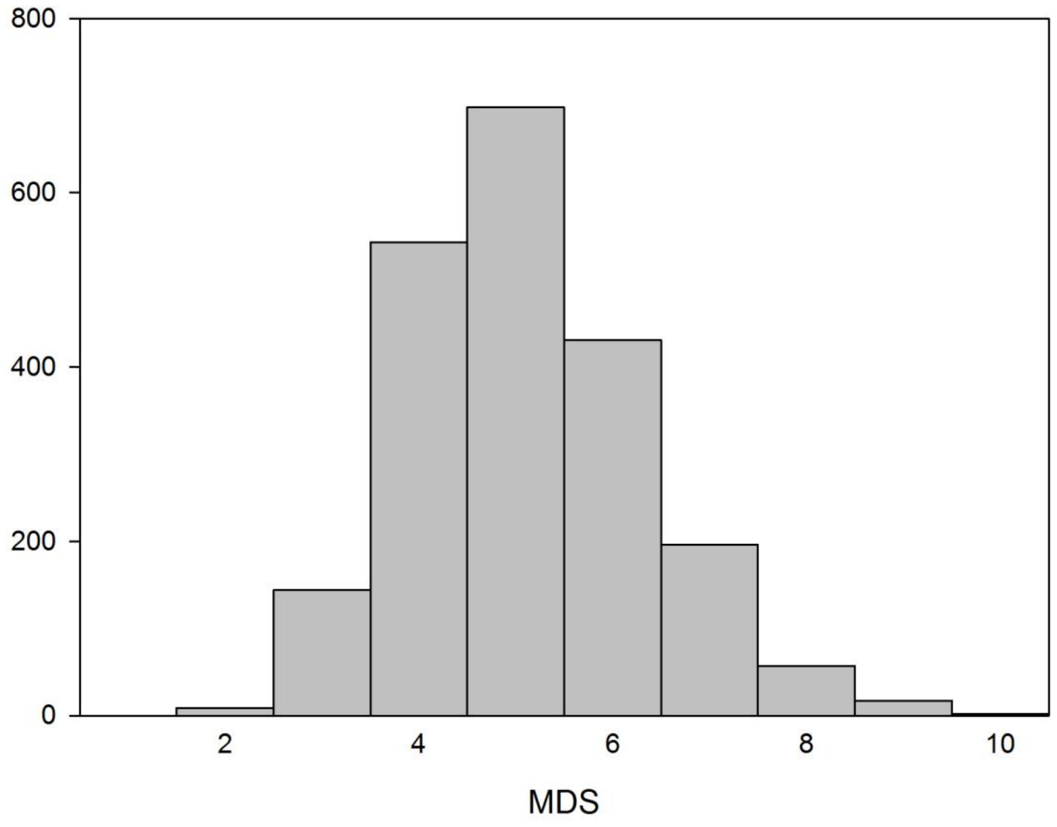

Supplement: Supplementary file 1 — supplementary table and figures [file 41598_2019_54938_MOESM1_ESM.pdf]
